# Investigation of the air permeability of fabric weaves to increase the wearing comfort of firefighter clothing and improve stab and cut protection

**DOI:** 10.1038/s41598-025-00264-3

**Published:** 2025-05-08

**Authors:** Rahel Heesemann, Sudhanshu Maurya, Rochak Rathour, Apurba Das, Thomas Gries

**Affiliations:** 1 Institut für Textiltechnik of RWTH Aachen University, Aachen, Germany; 2 Department of Textile and Fibre Engineering, Indian Institute of Technology Delhi, Delhi, India

**Keywords:** Firefighter, Personal protective clothing, Weaving, Wearing comfort, Protective performance, Physiology, Materials science

## Abstract

Firefighter protective clothing is composed of multiple layers, each serving distinct functions. The outer layer shields the user from fire, chemicals, cuts, body fluids, and water, while also permitting water vapour to escape. The middle membrane layer acts as a thermal and moisture barrier, preventing heat and liquid penetration but allowing vapour diffusion. The inner layer enhances thermal protection and wearer comfort. A nationwide German survey and risk analysis with different fire brigades identified a need for enhanced comfort, reduced physiological heat load, and improved protection against stabs and cuts. Enhanced tear resistance is one proposed method for increased stab and cut protection. Wearer comfort parameters include water vapour permeability, breathability, air permeability, efficient cooling and increased breathability of the protective clothing are crucial for comfort. Sweat is diffused through the jacket due to differing water vapour partial pressures inside and outside the jacket. Enhancing air permeability of the outer layer and reducing the water vapour transmission resistance across the entire layer structure improve cooling by lowering the external water vapour partial pressure, thus facilitating better sweat transport and heat dissipation. To increase breathability and stab- and cut protection, different fabric weaves for the outer layer of a firefighter´s jacket are produced and compared with each other. The Honeycomb and the Huck-a-back fabric achieve better properties than Twill 2/2 fabric used as standard.

## Introduction

The task of the fire brigade is to deal with dangerous situations such as fires or accidents as quickly as possible in order to minimise damage to people and property^1^. Firefighters are exposed to various hazards when fighting fires^2^. These hazards include, in particular, fire, smoke and heat (up to 800 °C). Specialised Personal Protective Equipment (PPE) is used to reduce the heat load on firefighters. This PPE also includes protective clothing for firefighters. This clothing not only offers protection against heat and flames, but also against moisture, chemicals and mechanical influences (e.g. abrasion, cuts)^3^.

**Fig. 1:**
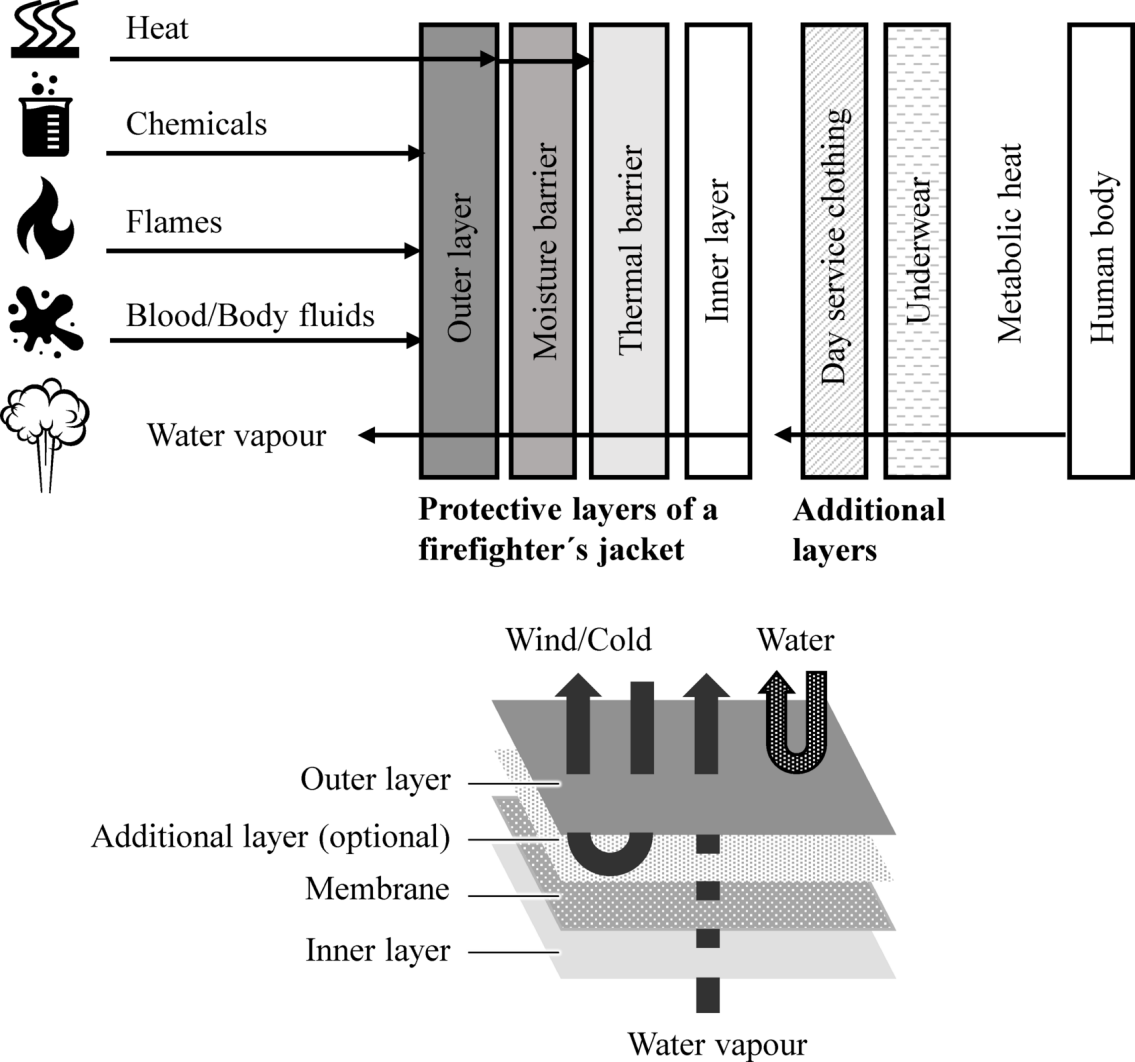
Different layer of a firefighter´s jacket^5,7^.

Firefighter protective clothing is made up of different layers^4^. Each layer fulfils a specific function^1^. The outer layer protects the user from fire, chemicals, cuts, body fluids (e.g. blood) and water. Another function of the outer layer is to allow water vapour to pass through (from the inside to the outside). The middle layer (membrane) protects against heat and the penetration of liquids (thermal barrier)^5,6^. On the other hand, the membrane enables the diffusion of water vapour to the outside (moisture barrier)^5,6^. The inner layer provides further thermal protection and increases wearer comfort. A schematic drawing of the different layers is shown in Fig. 1.

The individual layers are made from different fibres, materials and textile surfaces and patterns^1^. These materials can include the following:

- Inner layer: 93% m-aramid, 5% p-aramid, 2% antistatic (e.g. PA)^8^.
  - E.g. woven rip stop fabric, knitted fabric^9^.
- Membrane with backing non-woven: PTFE, non-woven 85% m-aramid, 15% p-aramid^10^.
- Outer fabric: 93% m-aramid, 5% p-aramid, 2% antistatic (e.g. PA) or 40% PBI, 60% p-aramid^11^.
  - E.g. Twill 2/2, Twill 3/1, Plain weave^9^.

Firefighter clothing is generally made of thick and heavy materials to ensure that firefighter clothing protects against various hazards. The thick and heavy clothing results in increased physiological strain^1,12^. In addition, the mobility of firefighters is restricted when they wear firefighting clothing together with equipment such as breathing protection. It is possible that the higher physiological load and the restricted mobility have an impact on the safety and health of the emergency personnel. Therefore, a compromise between safety and comfort is always necessary with firefighter clothing^7^^13^.

With the help of a German-wide user survey^14^ and a risk analysis with different fire brigades, it was determined that improved wearing comfort and a reduction in the physiological heat load of firefighters as well as increased protection against stabs and cuts are desired. One possibility for increased protection against stabs and cuts is a greater tear resistance. The parameters for determining wearing comfort include water vapour permeability, breathability, air permeability and fit. The aim is therefore to increase wearer comfort through cooling.

Increased wearer comfort can be achieved through increased breathability and more efficient cooling of firefighter protective clothing through improved sweat dissipation^6,12^. The human body releases sweat through the firefighter jacket into the environment^4^. This is a diffusion process. The diffusion process occurs due to different water vapour partial pressures inside and outside the firefighter jacket. To improve cooling, the water vapour partial pressure on the outside of the moisture barrier of the firefighter jacket is reduced. It is assumed that a reduction can be achieved by improving the air permeability of the outer layer and the water vapour permeability resistance of the entire layer structure^15^. An increased air flow transports sweat away more effectively and the water vapour partial pressure is reduced. This reduction leads to increased heat dissipation^4,16,17^.

Therefore, the aim is to improve wearing comfort as well as stab and cut protection via the outer layer of the firefighter jacket. The outer layer is the first to come into contact with the flames, chemicals, water or body fluids, making its structural integrity crucial for ensuring the inner layer performs optimally. The outer layer protects against mechanical influences such as abrasion, cuts and tears^3^.

## Materials and methods

A Huck-a-back and Honeycomb weave as well as a Twill 2/2 weave (Table 1) with a warp density of 28/cm and a weft density of 24/cm are produced for the outer layer of the overall system of a firefighter jacket.

The Huck-a-back weave has a repeat of 8 × 8. The structure of this weave is a combination of symmetrically arranged, longer floating yarns in two quadrants and a plain weave in the remaining two quadrants. The maximum length of the flotation is three yarns. The plain weave increases the tensile strength, while the longer flotation yarns increase the air permeability of the fabric^18–20^.

The size of the repeat of the Honeycomb weave is 8 × 8 and the structure appears similar to Honeycomb. Due to the symmetry of the weave, any forces that occur are distributed evenly. The weave consists of warp and weft yarns that are more firmly connected to each other than other warp and weft threads. Both the warp and weft threads can move freely on both sides of the fabric. The length of the floats in the Honeycomb fabric is up to three yarns. These flotations increase the air permeability^18–20^.

**Table 1:**
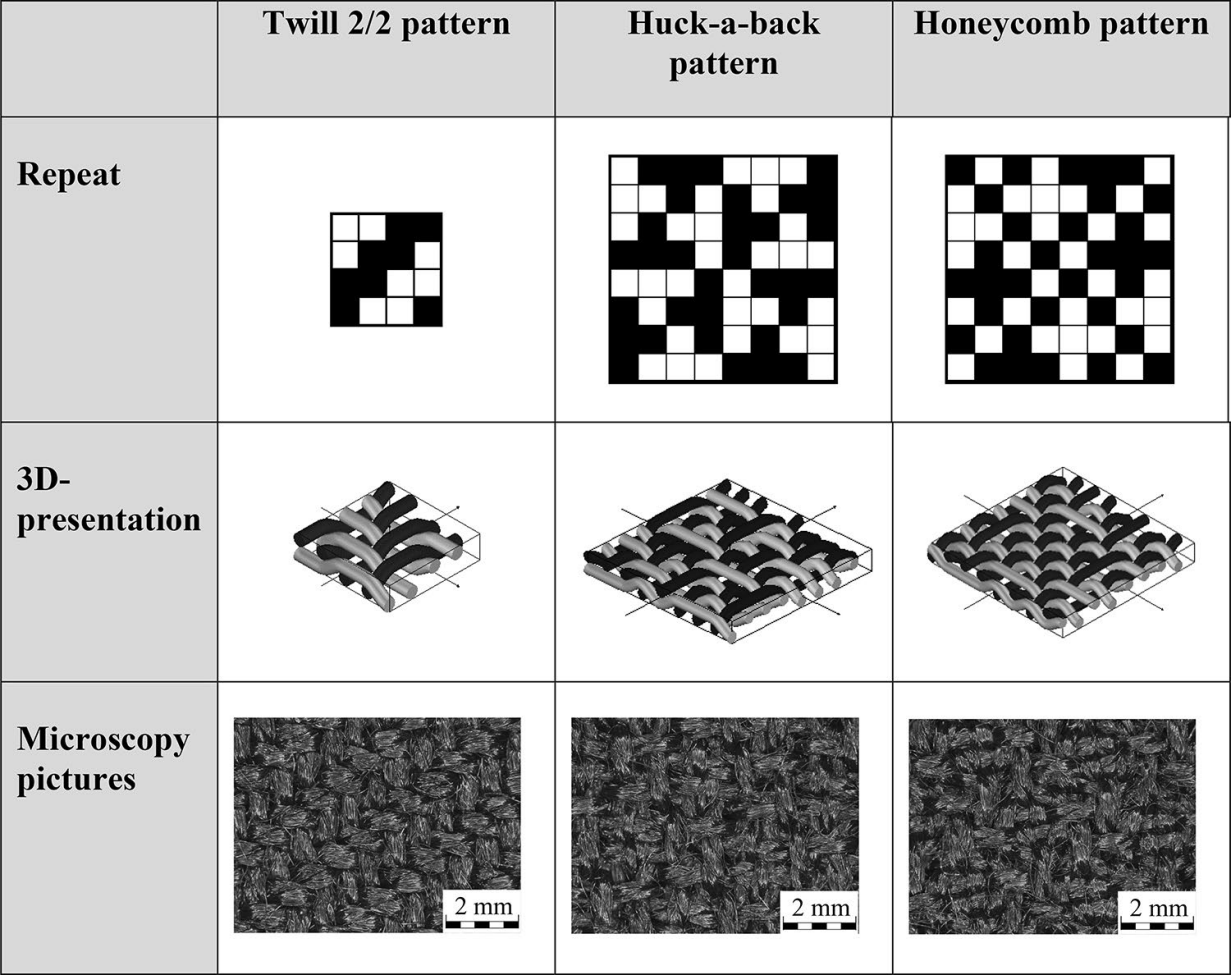
Twill 2/2, Huck-a-back and Honeycomb pattern.

**Fig. 2:**
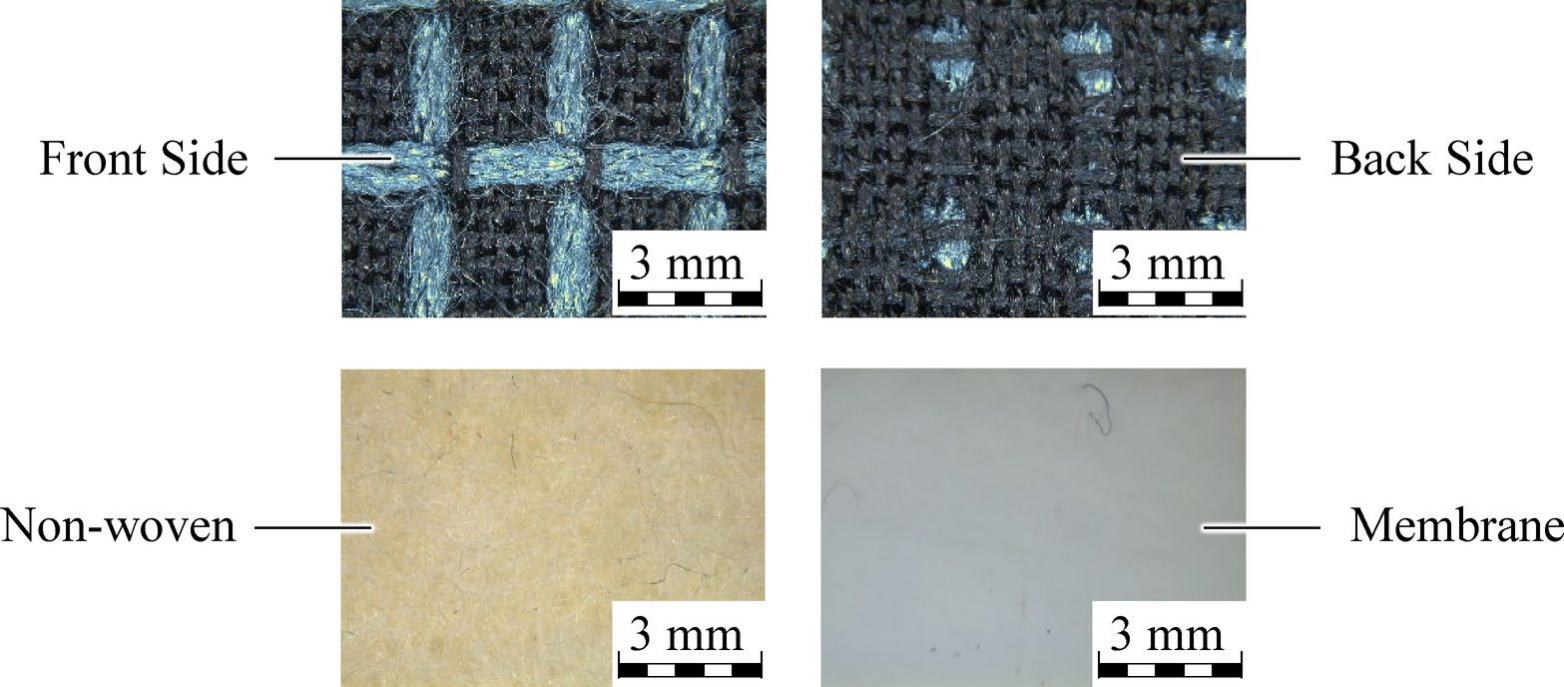
Above: inner layer; below: laminate with non-woven and membrane side.

The weaving machine used is the PTS 8/S C20 rapier weaving machine from Lindauer DORNIER GmbH, Lindau. The weaving width is 170 cm. The yarn material consists of 93% m-aramid, 5% p-aramid and 2% antistatic fibres. The laminate consists of a non-woven fabric made of 85% m-aramid and 15% p-aramid as well as a bi-component membrane based on polytetrafluoroethylene (PTFE). The laminate is used as standard in firefighter jackets. A ripstop fabric with a basis weight of 200 g/m^2^ is used for the inner layer. The open ripstop weave gives the inner layer a high level of air permeability. High air permeability is most important in the inner layer, as this is where there is nearest contact with the firefighter´s body. The fabric consists of 93% m-aramid, 5% p-aramid and 2% PA antistatic fibres. The inner layer and the laminate are shown in Fig. 2.

Following tests are carried out at standard climate:

- Physical performance.
  - Warp and weft density [1/cm] according to DIN EN 1049-2.
  - Area density [g/m^2^] according to DIN EN 12,127.
  - Thickness [mm] according to DIN EN ISO 5084.
  - Bulk density [kg/m^3^].
  - Porosity [%].
  - Tensile strength [N] according to DIN EN ISO 13,934.
  - Tear strength [N] according to DIN EN ISO 13937-2.
  - Abrasion resistance according to DIN EN ISO 12,947.
- Protective performance.
  - Heat Transfer Index HTI [s] according to DIN EN ISO 9151.
  - Radiant Heat Transfer Index RHTI [s] according to DIN EN ISO 6942.
  - Thermal resistance according to ISO 17493.
- Comfort performance.
  - Air permeability [mm/s] according to DIN EN ISO 9237.
  - Vater vapour permeability R_et_ [m^2^*Pa/W], Heat Transfer Resistance R_ct_ [m^2^*K/W], and Total Heat Loss Q_t_ [W/m^2^] with Integrated Sweating guarded-hotplate.

## Results

### Physical performance

The warp yarn density is between 29 1/cm and 31 1/cm. The weft density is 26 1/cm for the Huck-a-back and Honeycomb weave and 25 1/cm for the Twill 2/2 weave. The Twill 2/2 weave has the lowest warp and weft densities. The low yarn density is due to the shorter length of the flotation. Due to the increased number of crossing points, shrinkage is less than in fabrics with longer flotations. The grammages of the fabrics range from 195 g/m² to 207 g/m². The standard deviation is negligible with values ranging from 0.42 g/m² to 1.3 g/m². The Twill 2/2 weave has the lowest thickness with a value of 0.53 mm. The Honeycomb weave has a thickness of 0.79 mm. The Huck-a-back weave achieves a thickness of 0.69 mm. The porosity of the fabrics is determined using Eq. 1.

$$\:\text{Porosity=1-}\frac{\text{areal density of the fabric}}{\text{fibre}\text{ density}}\text{=1-}\frac{\frac{\text{grammage}}{\text{thickness}}}{\text{fibre}\text{ density}}$$

The total fibre density is 1.391 g/cm^3^. The fibre density is determined from the individual components and percentages of the yarn used:

- 93% m-aramid: 1.38 g/cm^3^.
- 5% p-aramid: 1.44 g/cm^3^.
- 2% antistatic fibre P140: 1.8 g/cm^3^.

The determination of the porosity is shown in Table 2.

**Table 2:** Determination of the porosity.

Pattern	Fibre density [kg/m^3^]	Thickness [m]	Grammage [kg/m^3^]	Porosity
**Huck-a-back**	1391	0,00069	0,199	0,793
**Honeycomb**		0,00079	0,207	0,812
**Twill 2/2**		0,00053	0,195	0,736

The Honeycomb pattern has the highest porosity with a value of 0.812 respectively. The Huck-a-back weave achieves a slightly lower porosity with a value of 0.793. The lowest porosity is achieved by the Twill 2/2 weave with a value of 0.736. In general, fabrics with higher porosity have lower thermal conductivity, as air is a better thermal insulator and therefore provides better thermal protection^21^.

**Fig. 3:**
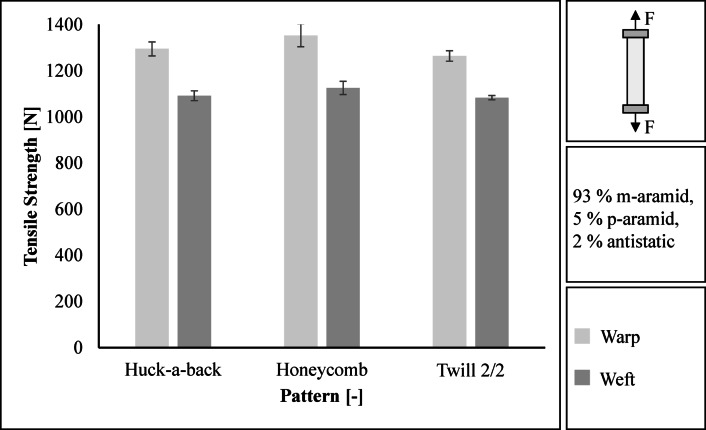
Results regarding tensile strength in warp and weft direction.

The tensile strength of the three fabrics is in a similar range (warp: 1264 N to 1353 N, weft: 1083 N to 1126 N), see Fig. 3. The Honeycomb fabric achieves the highest tensile strength in the warp and weft directions with values of 1353 N and 1126 N respectively. The standard deviation is 49.0 N in the warp direction and 28.9 N in the weft direction. The lowest tensile strength is achieved by the Twill 2/2 weave with 1264 N in the warp direction and 1083 N in the weft direction. The standard deviation is 22.6 N in warp and 9.4 N in weft.

**Fig. 4:**
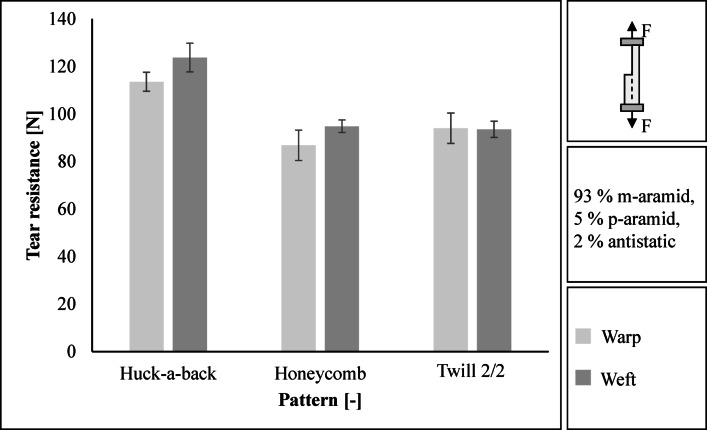
Results regarding tear resistance in warp and weft direction.

The Huck-a-back fabric achieves the highest tear resistance with 114 N in the warp direction and 124 N in the weft direction, see Fig. 4. The standard deviation is 4.0 N in the warp direction and 6.1 N in the weft direction. In the warp direction, the Honeycomb has the lowest tear resistance with a value of 87 N (standard deviation 6.4 N). In the weft direction, the 2/2 twill fabric has the lowest tear resistance at 94 N (standard deviation 3.4 N). The Huck-a-back weave achieves the highest tear resistance as the number of floats is greater than the Honeycomb and Twill 2/2 weave. Due to its tear resistance, the Huck-a-back weave offers the best protection against cuts and punctures.

According to the requirements of tenders from various German fire brigades, the fabrics must achieve a number of revolutions of at least 10,000 in the abrasion tests. Both the Twill 2/2 weave and the Huck-a-back and Honeycomb weaves achieve more than 20,000 cycles. As the requirements are met by twice the number of revolutions, the abrasion tests are cancelled after 20,000 revolutions.

### Protective performance

**Fig. 5:**
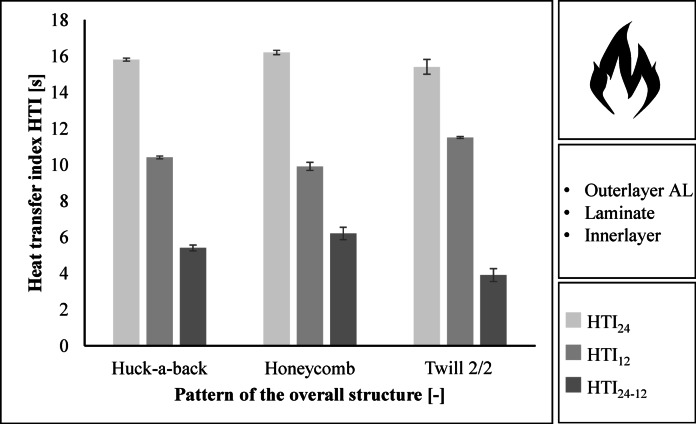
Results regarding the heat transfer index HTI when exposed to flames.

For a firefighter jacket of performance class 2, it is necessary to achieve a heat transfer index HTI_24_ value of at least 13.0 s and an HTI_24-12_value of at least 4.0 s^22^. The overall structure of the Huck-a-back fabric and the Honeycomb fabric exceed the target values. At 3.9 s, the total build-up of the twill-2/2 fabric does not achieve the HTI_24-12_ value. The Honeycomb fabric achieves the highest HTI_24_ and HTI_24-12_ values at 16.2 s and 6.2 s respectively, see Fig. 5.

**Fig. 6:**
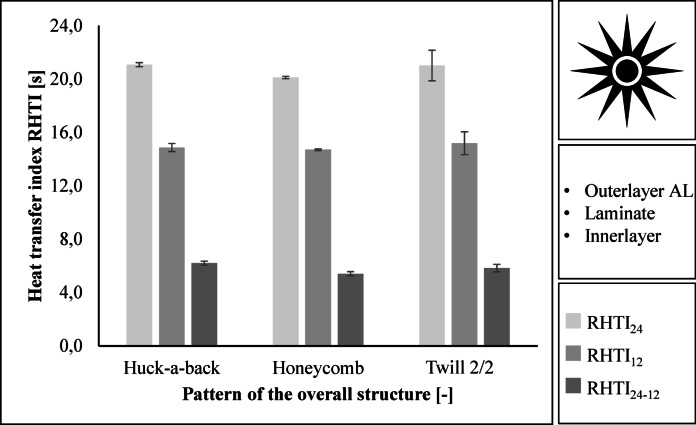
Results regarding the heat transfer index RHTI when exposed to radiation.

For a firefighter jacket in Performance class 2, an RHTI_24_ value of at least 18.0 s and an RHTI_24-12_value of at least 4.0 s is required^22^. The three assemblies meet these requirements. The RHTI values are close. The Huck-a-back construction achieves the highest heat transfer index with an RHTI_24_ value of 21.1 s and an RHTI_24-12_ value of 6.2 s. The Honeycomb construction achieves the lowest RHTI_24_ value of 20.1 s and the lowest RHTI_24-12_ value of 5.4 s. Figure 6 shows the results.

To determine the thermal resistance, the fabric structures are placed in a hot air oven at 185 °C for 5 min. The shrinkage is then determined. The shrinkage must not exceed 5%. The fabrics must neither ignite nor melt. The sample size is 16 × 16 cm. Three tests are carried out per layer structure. Shrinkage greater than 5% is not detected. Table 3 shows the samples before and after testing the thermal resistance.

**Table 3:**
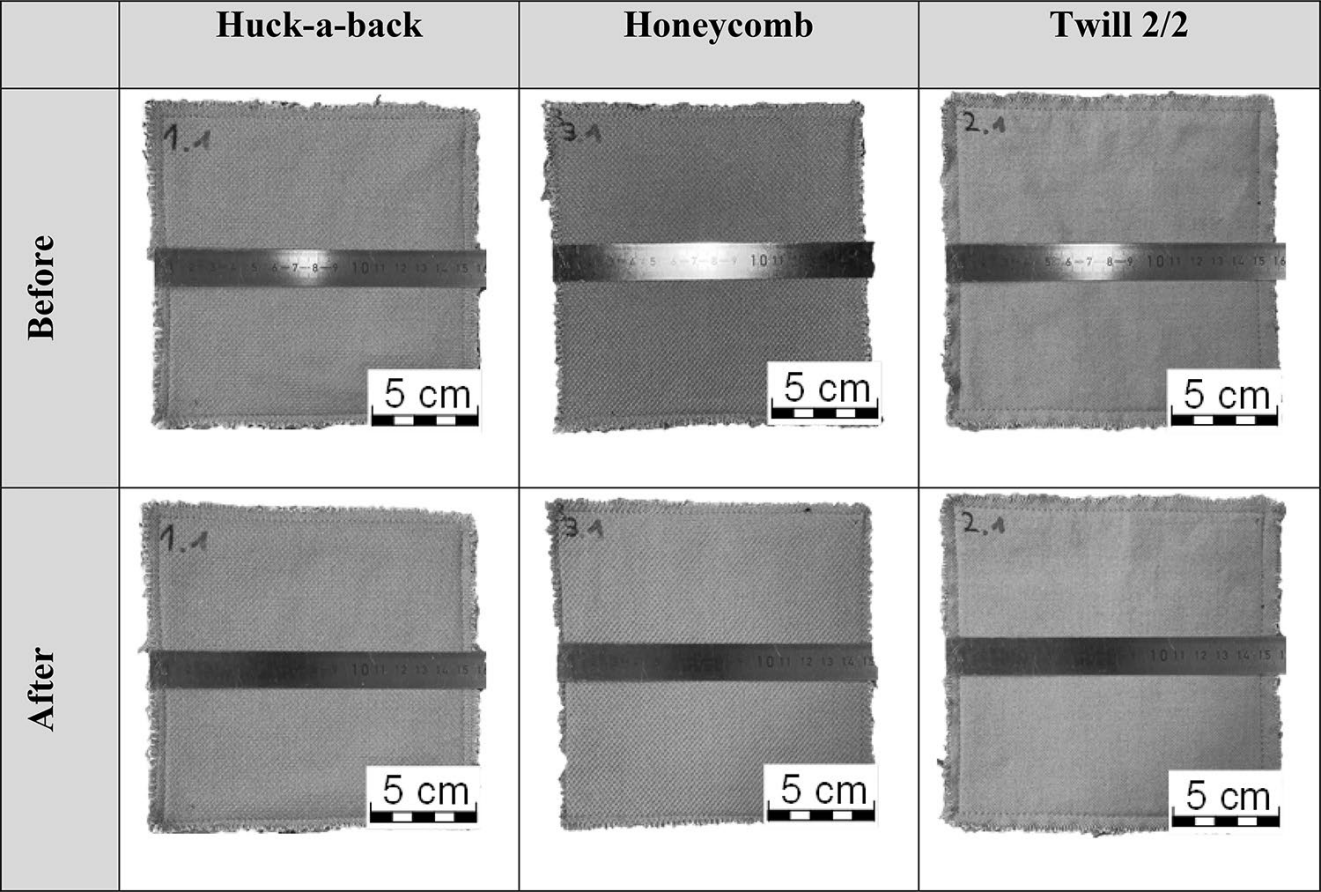
Results regarding thermal resistance according to ISO 17493, fabric before and after the hot air oven at 185 °C for 5 min.

### Comfort performance

The Huck-a-back fabric achieves the highest air permeability of 543 mm/s for the outer layer of the firefighter jacket. The Honeycomb fabric achieves an air permeability of 424 mm/s. The lowest value is 210 mm/s for the Twill 2/2 fabric. The laminate is not air permeable. However, the laminate is breathable. In the case of firefighter clothing, the breathability of the whole structure is important. DIN EN 469 does not require air permeability, but water vapour transmission resistance R_et_according to DIN EN ISO 11092^22^.

**Fig. 7:**
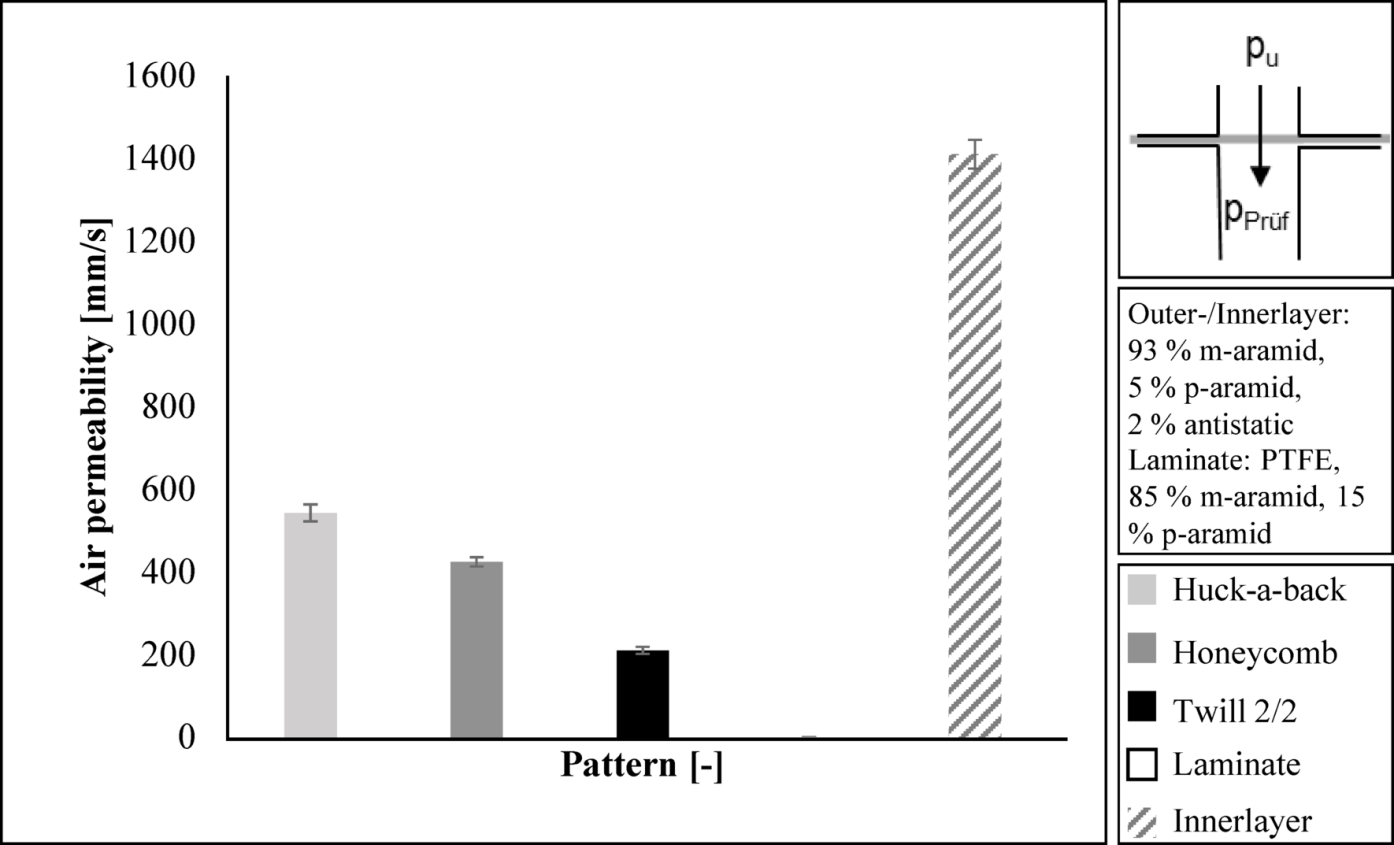
Results regarding the air permeability.

The inner layer has an air permeability of 1410 mm/s. This is approximately three times the air permeability of the Huck-a-back fabric. The inner layer is directly next to the body. It is therefore necessary for the inner layer to have the highest air permeability in order to transport perspiration. The results are given in Fig. 7.

The air permeability of the entire layer structure is low with values between 0.41 mm/s and 0.97 mm/s. The standard deviation is high at 0.20 to 0.37 mm/s. A comparison of the individual layer structures is therefore not meaningful. The reason for the low air permeability and high standard deviation is the air-impermeable laminate.

**Fig. 8:**
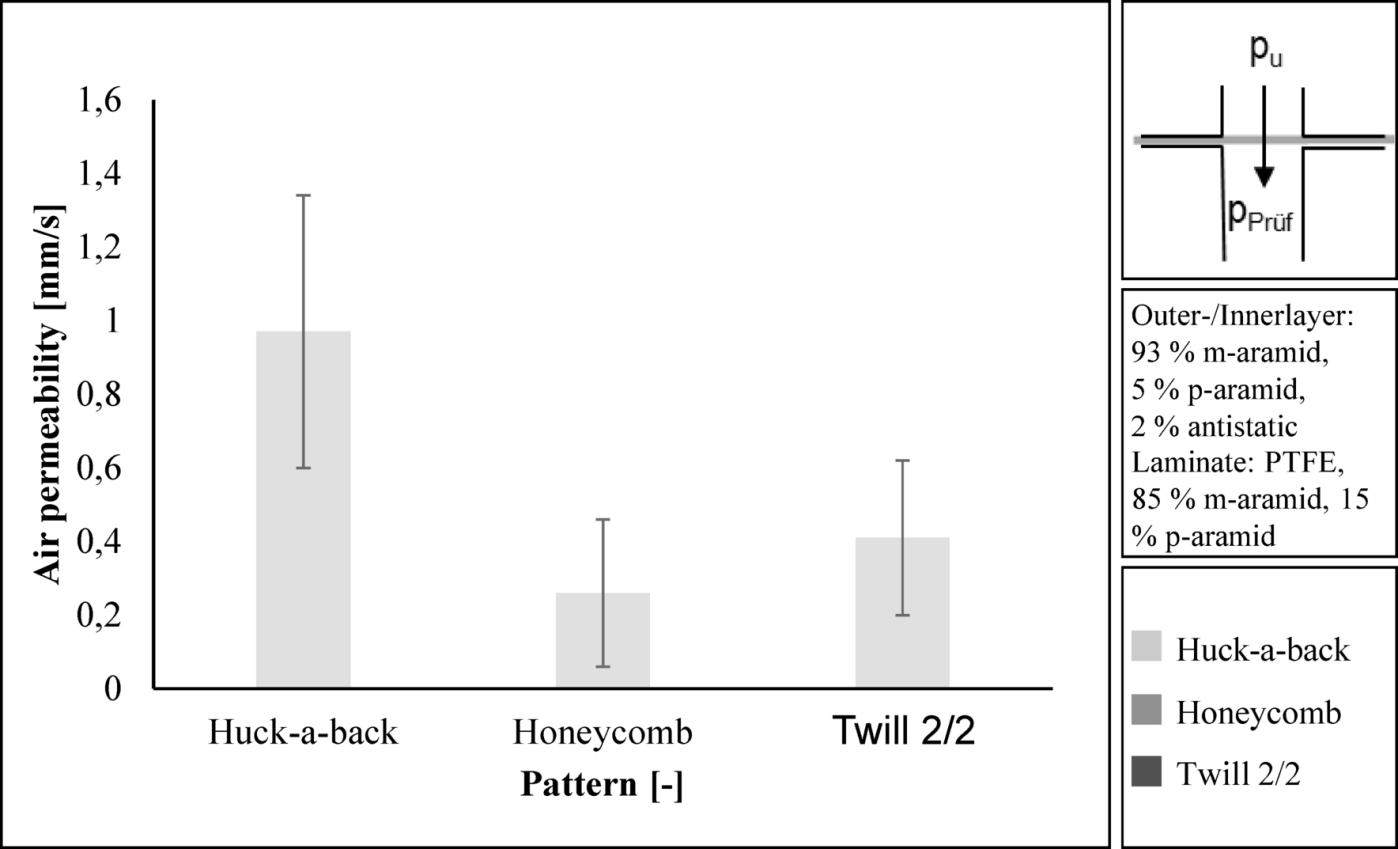
Air permeability of the entire layer structure.

Figure 8 shows the air permeability of the entire structure of the firefighter jacket.

Water vapour resistance R_et_, thermal resistance R_ct_ and total heat loss Q_t_are determined at the Department of Textile and Fibre Engineering, Indian Institute of Technology (IIT) Delhi, Delhi, India. The test is carried out on an Integrated Sweating Guarded Hotplate (iSGHP) from Thermetrics LLC, Seattle, Washington, USA, according to ASTM F 1868 C^23^. Both the individual layers and the overall structure of the layers are tested. The water vapour resistance R_et_ is determined using Eq. 1^24^ :

1 $$\:{\text{R}}_{\text{et}}\text{=}{\text{R}}_{\text{ef}}\text{-}{\text{R}}_{\text{et0}}\text{=}\frac{{\text{(p}}_{\text{m}}\text{-}{\text{p}}_{\text{a}}\text{)*A}}{\text{H-}{{\Delta}{H}}_{\text{e}}}\text{-}{\text{R}}_{\text{et0}}$$

- p_m_: Saturation water vapour partial pressure at the measuring head surface, in Pa, at temperature T_m_.
- p_a_: Water vapour partial pressure in the air in the test room, in Pa, at temperature T_a_.
- A: Area of the measuring head, in m^2^.
- H: Heating power supplied to the probe head, in W.
- ∆H_e_: Correction element for the heat output when measuring the water vapour transmission resistance R_et_.
- R_et0_: Device constant, in m^2^ *Pa/W, when measuring the water vapour transmission resistance R_et_.
- T_a_: Air temperature in the test room, in °C (Degrees Celsius).
- T_m_: Temperature of the probe head, in °C.

**Fig. 9:**
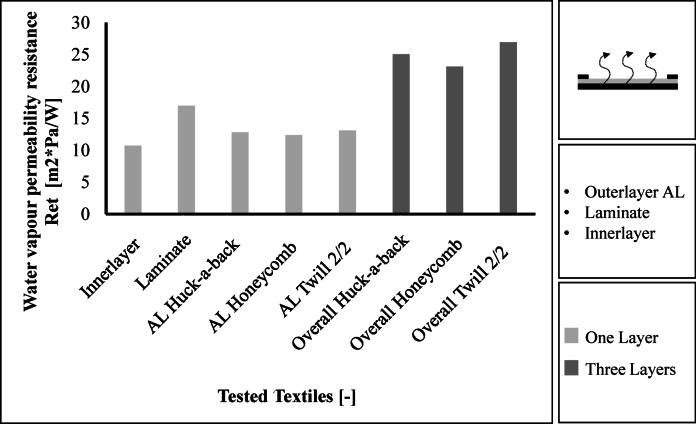
Results regarding the water vapour transmission resistance.

The results for water vapour resistance are shown in Fig. 9. The inner layer has the lowest water vapour resistance with a value of 10.7 m^2^*Pa/W. The laminate has the highest water vapour resistance with a value of 17.0 m^2^*Pa/W. The outer layers have values between 12.4 m^2^*Pa/W and 13.1 m^2^*Pa/W, while the Twill 2/2 fabric having the highest water vapour transmission resistance and the Honeycomb fabric having the lowest. The 2/2 twill fabric also achieves the highest water vapour transmission resistance of 26.9 m^2^*Pa/W in the overall layer construction. The Huck-a-back fabric achieves a water vapour transmission resistance of 25.1 m^2^*Pa/W and the Honeycomb fabric achieves a value of 23.1 m2*Pa/W in the total layer structure. The three layer structures therefore meet the requirements for a maximum water vapour transmission resistance of 30 m^2^*Pa/W.

The thermal resistance R_ct_ is determined using Eq. 2^24^:

2 $$\:{\text{R}}_{\text{ct}}\text{=}{\text{R}}_{\text{cf}}\text{-}{\text{R}}_{\text{ct0}}\text{=}\frac{{\text{(T}}_{\text{m}}\text{-}{\text{T}}_{\text{a}}\text{)*A}}{\text{H-}{\Delta{H}}_{\text{C}}}\text{-}{\text{R}}_{\text{ct0}}\:$$

- T_m_: Temperature of the probe head, in °C.
- T_a_: Air temperature in the test room, in °C (Degrees Celsius).
- A: Area of the measuring head, in m^2^.
- H: Heating power supplied to the probe head, in W.
- ∆H_c_: Correction element for the heat output when measuring the thermal resistance R_ct_.
- R_ct0_: Device constant, in m^2^*Pa/W, when measuring the thermal resistance R_ct_.

**Fig. 10:**
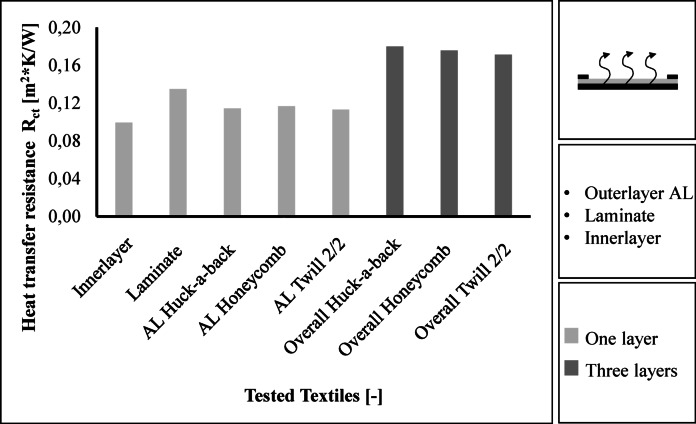
Results regarding the thermal resistance.

The results for heat transfer resistance are shown in Fig. 10. The inner layer has the lowest thermal resistance with a value of 0.099 m^2^*K/W. The laminate achieves the highest thermal resistance with a value of 0.135 m^2^*K/W. The three outer layers achieve values between 0.113 and 0.117 m^2^*K/W, with the Twill 2/2 fabric having the lowest thermal resistance. The Twill 2/2 fabric also achieves the lowest thermal resistance of all the layers with a value of 0.171 m^2^*K/W. The Huck-a-back fabric achieves a value of 0.178 m^2^*K/W and the Honeycomb fabric achieves a thermal resistance of 0.176 m^2^*K/W.

The total heat loss Q_t_ is determined from the averaged intrinsic thermal resistance R_cf._ (Eq. 5) and the averaged intrinsic water vapour transmission resistance R_ef_ (Eq. 4), see Eq. 5.

3 $${\text{R}}_{\text{cf.}}={\text{R}}_{\text{ct}}-{\text{R}}_{\text{ct0}}$$

4 $${\text{R}}_{\text{ef}}={\text{R}}_{\text{et}}-{\text{R}}_{\text{et0}}$$

5 $$\:{\text{Q}}{\text{t}}\text{=}\frac{{10\,^\circ}\text{C}}{{\text{R}}{\text{cf}}\text{+0,04}}\text{+}\frac{\text{3,75}\text{kPa}}{{\text{R}}_{\text{ef}}\text{+0,0035}}$$

**Fig. 11:**
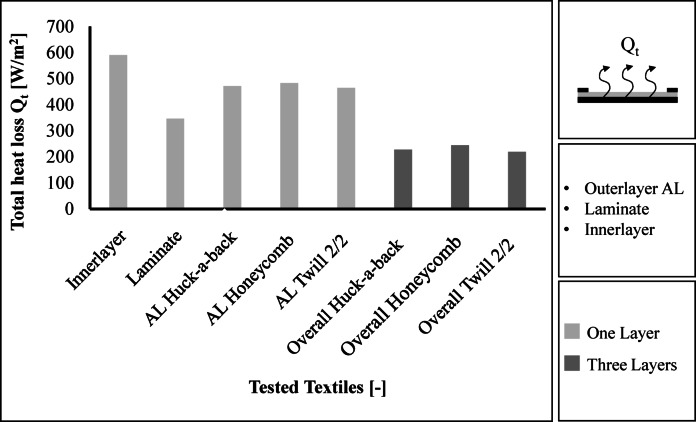
Results regarding the total heat loss.

For the test, R_ct0_ is 0.0759 m^2^*K/W and R_et0_ is 5.978 m^2^*Pa/W. The total heat loss results are shown in Fig. 11. The total heat loss is similar for the different constructions. The Twill 2/2 overall structure has the lowest total heat loss at 465 W/m^2^. The Honeycomb overall structure achieves a total heat loss of 483 W/m^2^. A higher total heat loss means a higher release of heat. The Honeycomb fabric therefore achieves the highest breathability.

## Discussion

### Physical performance

Due to the high proportion of plain weave and the alternating floating, the Honeycomb fabric achieves the highest tensile strength. The maximum tensile strength of the fabric is limited by the strength of the yarns used^25^. The tensile strength of a fabric increases with yarn density. The shorter the flotation in a fabric, the higher the tensile strength. Friction between the warp and weft yarns transmits forces. In addition, an even distribution of interlacing points leads to higher tensile strength. A plain weave will therefore have the highest tensile strength^26,27^.

The Huck-a-back fabric has the highest tear resistance compared to Twill 2/2 and Honeycomb fabric due to its longer floatation. The three patterns fulfil the minimum requirements for tear resistance of 50 N in the warp and weft direction in accordance with DIN EN 469 and DIN EN ISO 13937-2^22,28^. They even exceed the values of currently used outer layers. The average values in the warp and weft direction, for example, are 150 to 180 N^29^. Due to its high tear strength, the Huck-a-back weave offers the best protection against cuts and punctures. Tear resistance is reduced by higher warp and weft densities. Longer flotation in a fabric increases the tear resistance. The reason for the higher tear resistance of a loose fabric is that the individual yarns can move and form groups. For example, plain fabrics have the lowest tear resistance because the warp and weft yarns cannot move^9,30–32^.

The abrasion resistance of a material is influenced by the twist of the yarn and the contact surface between the yarn and the abrasive^18^. A larger contact area results in higher abrasion resistance. In a highly twisted yarn, the fibres are well bonded. This makes it more difficult to pull the fibres out of the yarn. Abrasion resistance is improved. On the other hand, the contact surface between the yarn and the abrasive is greater with a low twist yarn than with a high twist yarn. Therefore, due to the increased contact surface between the yarn and the abrasive, even a low yarn twist can increase abrasion resistance. Studies by Kaynak and Topalbekiroglu have shown that long flotation and low yarn twist reduce abrasion resistance. The lower abrasion resistance is due to the long flotation, which increase the flexibility of the yarn. Due to the deformation of the yarn, the fibres hold together less well and the abrasion resistance decreases^18,32,33^.

### Protective performance

The heat transfer index for flames (HTI) and jets (RHTI) is determined by the time it takes for a temperature rise of 12 °C and 24 °C to occur. In addition, the time is calculated from the difference between a temperature rise of 24 °C and 12 °C. The time for a temperature rise is increased by increased filament density and longer flotation^34,35^.

Thermal conductivity or contact heat is highest for plain weave fabrics. Thermal conductivity increases as the amount of flotation in a fabric decreases. The thermal conductivity of a fabric also increases as the yarn density increases. For a firefighter jacket, low thermal conductivity from the outside to the inside is desirable^30,36^.

The HTI and RHTI values of the three fabrics are in the same range, with the HTI value of the Honeycomb fabric being the highest. However, the RHTI value is lowest for the Honeycomb fabric. It is therefore not possible to make a clear statement. Further tests should be carried out in the future to determine the dependency between the heat transfer index of radiation and flames.

### Comfort performance

As all three fabrics were produced with the same material, the values regarding the heat transfer resistance do not differ significantly. The heat transfer resistance of a fabric depends on the material. For cotton fabrics, thermal resistance is increased by long flotation, as cotton is a hollow fibre^37,38^. For example, a 2/2 twill cotton fabric has a lower heat transfer resistance than a 5/3 Twill cotton fabric^38,39^. For solid fibres with a round cross section (e.g. aramid fibres) the heat transfer resistance decreases with long flotation. An aramid fabric with a 3/1 twill weave will therefore have a lower heat transfer resistance than one with a 2/2 twill weave^21^. The heat transfer resistance of a fabric decreases as the porosity of the fabric decreases because there is less space for air to be trapped. The lower the heat transfer resistance of a fabric, the more suitable it is for high temperatures^21^. For fabrics with flame retardant yarns (e.g. aramid), the heat transfer increases as the yarn density increases. The reason for this is that as the amount of yarn increases, so does the thermal behaviour of the fabric^21^.

As the Huck-a-back and Honeycomb fabrics have more flotation than the twill fabric, the air permeability is higher. The water vapour transmission resistance is lower for both fabrics. The more porous the fabric and the longer flotation, the higher the air permeability and water vapour resistance. Air permeability and moisture vapour transmission resistance are reduced by increasing the yarn density in the fabric^21^.

## Conclusions

Long flotation is advantageous for tear resistance and air permeability. However, the increased mobility provided by longer flotation reduces the abrasion resistance and tensile strength of the fabric. Heat conduction is reduced with long flotation. Thermal resistance depends on the fibre material. However, since an aramid mixtures is chosen, it can be assumed that the thermal resistance will decrease with longer flotation^21^. To increase wearer comfort, the aim is to develop a fabric with high air permeability and a water vapour transmission resistance of ≤ 30 m^2^Pa/W. A compromise is therefore sought to achieve the longest possible flotation without unduly compromising abrasion resistance and tensile strength.

1. short flotation increases tensile strength.
2. long flotation increases tear resistance.
3. groups of yarns close together increase tear resistance.
4. large contact surface increases abrasion resistance.
5. short flotation increase abrasion resistance.
6. long flotation reduces heat conduction (increase insulation).
7. long flotation increases thermal resistance.
8. long flotation increases air permeability.
9. long flotation reduce resistance to water vapour transmission.

In the overall layer structure, high air permeability has no influence, as the laminate (middle layer) is not permeable to air^10^. A low water vapour transmission resistance is also relevant for higher breathability^39^. Both the Honeycomb and the Huck-a-back overall structure achieve a lower water vapour transmission resistance than the Twill 2/2 fabric. The Honeycomb overall structure achieves the lowest water vapour transmission resistance. Therefore, it is possible that there is a correlation between a high air permeability and a low water vapour resistance. However, this must be proven in further investigations.

The Huck-a-back fabric achieves the highest tear resistance, which provides increased protection against stabs and cuts. The Honeycomb and the Huck-a-back overall structure fulfil the requirements for thermal protection as well as the other physical requirements and exceed the properties of the Twill 2/2 fabric.

## Data Availability

The raw data supporting the conclusions of this article will be made available by the authors on request. Please contact Rahel Heesemann: rahel.heesemann@ita.rwth-aachen.de.
